# Systematic screening and focused evaluation for veno-occlusive disease/sinusoidal obstructive syndrome (VOD/SOS) following allogeneic stem cell transplant is associated with earlier diagnosis and prompt institution of defibrotide treatment

**DOI:** 10.3389/frtra.2022.996003

**Published:** 2022-11-08

**Authors:** Daniele Avenoso, Michelle Kenyon, Varun Mehra, Pramila Krishnamurthy, Austin Kulasekararaj, Shreyans Gandhi, Francesco Dazzi, Mili Naresh Shah, Henry Wood, Ye Ting Leung, Alicia Eaton, Sandra Anteh, Maria Cuadrado, Madson Correia de Farias, Christienne Bourlon, Diana Oana Dragoi, Prudence Hardefeldt, Antonio Pagliuca, Victoria Potter

**Affiliations:** Department of Haematological Medicine, King's College Hospital NHS Foundation Trust, London, United Kingdom

**Keywords:** VOD, defibrotide, myeloablative allogeneic hematopoietic cell transplantation, reduced intensity allogeneic stem cell transplant, SOS (sinusoidal obstruction syndrome)

## Abstract

Sinusoidal obstructive syndrome (SOS), also known as hepatic veno-occlusive disease (VOD), is a potentially life-threatening complication following haemopoietic stem cell transplantation (HSCT). The availability of new drugs for malignant hematological conditions has allowed more patients to be eligible for allogeneic haematopoietic stem cell transplants, which has translated into a significant proportion of transplant patients having multiple risk factors for VOD/SOS. Based on these considerations, we undertook a dedicated weekly VOD/SOS ward round, aiming to facilitate early diagnosis of VOD/SOS and pre-emptively identify patients at risk, where a careful evaluation of differential diagnosis is essential. Herein, we present the results of our VOD/SOS ward round; between September 2020 and April 2022, 110 consecutive patients were evaluated in a focused VOD/SOS ward round. From the 110 patients, 108 had undergone HSCT and had at least one known risk factor for developing VOD/SOS. The median number of risk factors present in the VOD/SOS group and non-VOD/SOS group was five (range: three to six) and three (range: zero to seven), respectively. Late-onset VOD/SOS was diagnosed in 45% of our patients. The early identification of patients with multiple risk factors for VOD/SOS allowed an earlier diagnosis and the administration of defibrotide on the same day of diagnosis, which was two days earlier than our previous experience prior to the implementation of this protocol.

## Introduction

Sinusoidal obstructive syndrome (SOS), also known as hepatic veno-occlusive disease (VOD), is a potentially life-threatening complication following haemopoietic stem cell transplantation (HSCT) (1).

The availability of novel agents in the treatment of high-risk hematological malignancies as well as reduced-intensity conditioning (RIC) has led to increased numbers of HSCT-eligible patients (2, 3) which has translated into a significant proportion of transplant patients having multiple risk factors for VOD/SOS. As previously reported, identification of patients at risk, prompt diagnosis of VOD/SOS, and the administration of defibrotide therapy before the onset of multiorgan failure can be lifesaving (4, 5). Based on these considerations, we undertook a dedicated weekly VOD/SOS ward round, aiming to facilitate early diagnosis of VOD/SOS and pre-emptively identify patients at risk, where a careful evaluation of differential diagnosis is essential.

## Rationale

A review of VOD/SOS cases during the two-year period between July 2018 and June 2020 identified our approach to identification of patients at risk, VOD/SOS diagnosis, and treatment strategies as areas for service improvement. In July 2020 it was decided that a systematic and standardized approach to VOD/SOS be developed through a dedicated ward round to identify patients at risk for the complication, to support the management of it, and to increase awareness of VOD/SOS for all members of the HSCT service team at King's College Hospital, London.

A standard approach was designed as described below, together with uniform documentation of the findings.

A pilot ward round was delivered over a period of 4 weeks to clarify its feasibility and safety. A review of the process showed its potential value because of the systematic and clear method of documentation regarding VOD/SOS and regular discussion about it. For this reason, the dedicated ward round subsequently became part of routine practice at our centre.

### Patient selection

Allogeneic stem cell transplant recipients underwent a weekly formal assessment for VOD/SOS until discharge. This was a supplemental, independent evaluation to the daily ward round. In the event of re-admission, assessment continued until at least day 60. A total of 110 consecutive patients who had received a transplant between September 2020 and April 2022 were evaluable for this analysis. Demographics, transplant details, and VOD/SOS characteristics are summarized in Table 1.

**Table 1:** Demographic of the study population and VOD features.

**Patients characteristics**	**N (%)**
**Number of patients** (*n*, %)	110 (100)
Female	46 (40)
Male	64 (60)
Age, years (median, range)	57 (17–73)
**Diagnosis**	
Aplastic anemia	12 (10)
Acute myeloid leukemia	42 (38)
B-cell acute lymphoblastic leukemia	8 (7)
Chronic myeloid leukemia	8 (7)
Chronic myelomonocytic leukemia	2 (1)
Hodgkin lymphoma	3 (2)
Myelodysplastic syndrome	18 (16)
Myelofibrosis	8 (7)
T-cell lymphoma	2 (1)
Sickle cell disease (adult)	3 (2)
Pure red cell aplasia	2 (1)
**Conditioning intensity**	
Myeloablative	41 (37)
Reduced intensity	65 (70)
Non-myeloablative	4 (3)
**Myeloablative Features**	
High dose TBI**	3 (2)
High dose Bu*	38 (34)
T-replete	6 (5)
T-deplete (ATG or Campath)	104 (95)
**Donor**	
Full-matched sibling	20 (18)
Full-matched unrelated	41 (37)
Mismatched unrelated	43 (39)
Haploidentical sibling	6 (5)
Second allograft	9 (8)
Active disease or beyond CR2	42 (38)
**VOD features – total n**	12
Days to onset (range)	14 (8–44)
Late-onset VOD/SOS	5
Risk factors present (median, range)	5 (3–7)
Severe	11
Very severe	1
Intensive care admission	1
Death	1

High-dose busulfan is defined if the total dose is > 6.4 mg/Kg; TBI = total body irradiation; high-dose TBI is defined if the dose is ≥ 8 Gy fractionated; CR2 = second complete remission. Patients received either myeloablative conditioning (MAC) or reduced-intensity conditioning (RIC) based on disease characteristic and performance status. All the patients (bar the four haploidentical procedures) had T-cell depletion *in vivo* with ATG (total 5 mg/Kg) or Campath (total dose 60 mg) based on disease-specific conditioning protocol. GVHD prophylaxis was ciclosporin single agent with a therapeutic level of 150–200 ng/ml for 56 days tapered in absence of GVHD aiming for cessation of immunosuppressive therapy by D100. For aplastic anemia, a therapeutic cyclosporine level of 250–350 ng/ml was maintained until 12 months post-transplant. Haploidentical stem cell transplant received standard GVHD prophylaxis with post-transplant cyclophosphamide, mycophenolate, and tacrolimus. Only two transplants from matched unrelated donors had post-transplant cyclophosphamide as GVHD prophylaxis. HLA typing is performed utilizing both Third Generation Sequencing (TGS) and Next Generation Sequencing (NGS) techniques. Mismatched donors were considered as <12/12 match. *and **recall the definitions for high dose Bu and high dose TBI.

### VOD/SOS ward round

**Figure 1:**
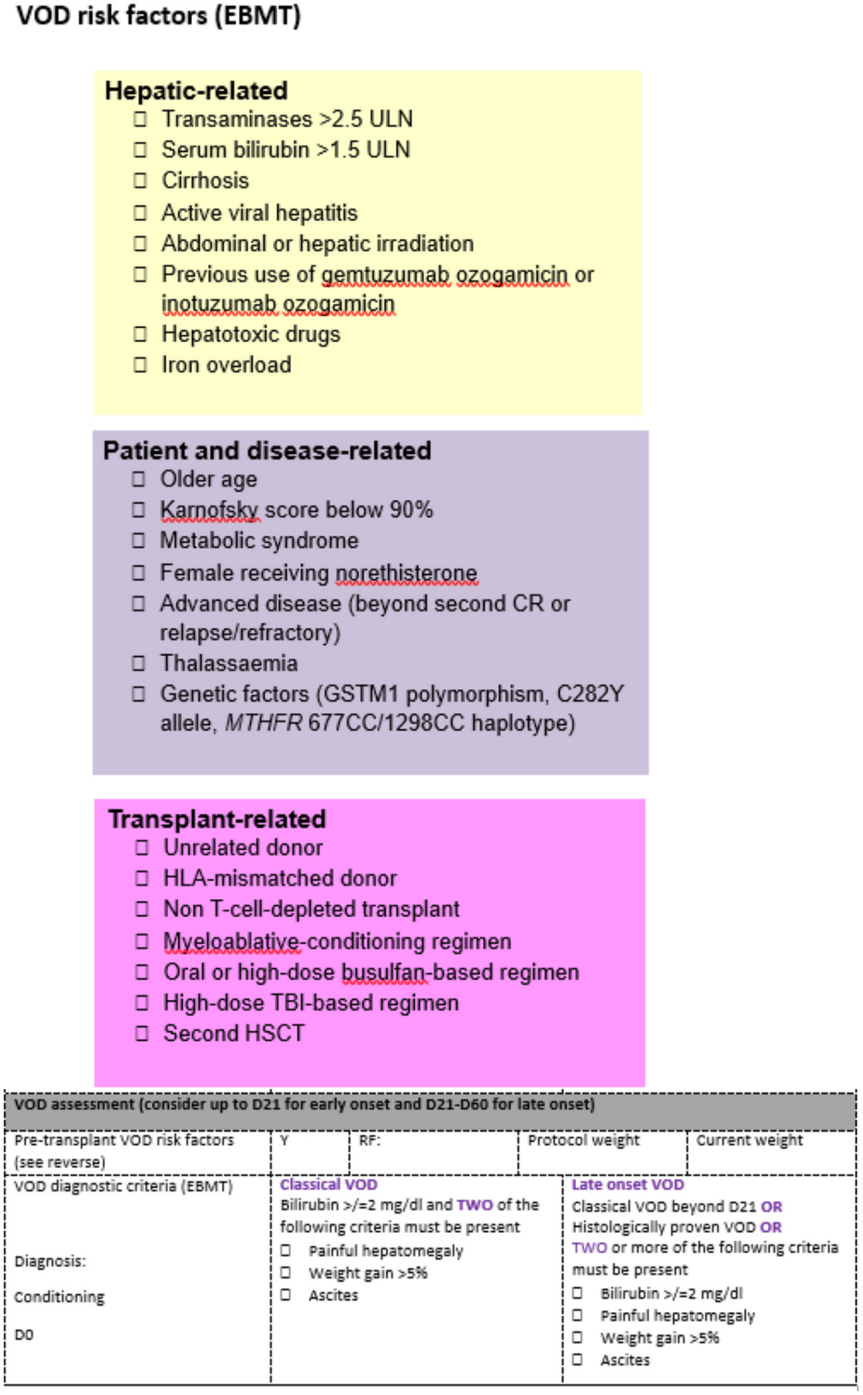
VOD/SOS ward round form. The colored parts highlight the risk factors present for each patient and are assessed before the admission. The VOD/SOS assessment form is the tool that allows summarization of the clinical evaluation.

Known pre-transplant risk factors are documented on a dedicated VOD/SOS assessment sheet on admission, to aid differential diagnosis and enable the correct grading when a patient with clinical signs and symptoms of VOD/SOS is identified (Figure 1). The weekly VOD/SOS assessment (in addition to the regular one from the ward attending team) is performed by a dedicated team, consisting of a consultant hematologist and consultant nurse in stem cell transplantation, to provide a consistent approach in assessments over time. The day before the dedicated ward round, the forms containing the risk factors are reviewed to ensure correct documentation of all risk factors. At the time of joint evaluation, the following clinical parameters are evaluated: performance status, vital signs, current body weight and comparison with pre-transplant weight, value of bilirubin, and renal function. Evaluation of the drug chart is also conducted to assess potential drug interaction that could result in raised bilirubin. The physical examination aims to evaluate the presence of right upper quadrant pain, hepatomegaly, and ascites. Once the joint clinical evaluation is completed, the risk factors are reviewed again, and we assess if the patient meets the diagnostic criteria for VOD/SOS.

All findings are written in the clinical notes, and there is also an oral handover to both nurse and medical ward attending team to share the findings from the focused evaluation. If during the ward round a patient is identified to be at risk for VOD/SOS, the ward team will perform daily monitoring of the patient to avoid delays in diagnosis.

### VOD/SOS evaluation and diagnostic criteria and management

The previously described diagnostic criteria were used for diagnosis of both classical and late-onset VOD/SOS, and were based on guidelines published by the European Society for Blood and Marrow Transplantation (EBMT) (6). The treatment is based on intravenous defibrotide at standard dose (25 mg/kg/day) for 21 days alongside enhanced supportive care with adjusted transfusion threshold (aim for platelets > 25 × 10^9^ /L, hemoglobin > 85 g/L), twice daily weight measurement, strict fluid balance, and early consultation with intensive care and liver physicians.

## Results

### Risk factors identified

Out of 110 patients, 108 underwent HSCT with at least one known risk factor for developing VOD/SOS. The median number of risk factors present in the VOD/SOS group and non-VOD/SOS group were five (range: three to six) and three (range: zero to seven), respectively. Within the VOD/SOS group, 10 out 12 patients received myeloablative conditioning (MAC), and two patients with heavily pre-treated lymphoid neoplasms received RIC; 77% of the patients had received cells from a mismatched unrelated donor, and the remaining patients had received cells from a fully matched unrelated donor. Non-VOD/SOS patients received mainly RIC (67/98), and the remaining patients received MAC (31); the majority had received stem cells from a mismatched unrelated donor (37/98), and the remainder comprised of those who had received stem cells from a fully matched unrelated donor (36/98), a matched sibling (19), or a haploidentical donor (6). Thirty-eight percent of the subjects had active disease or were beyond CR2 at the time of transplant. Table 2 shows the frequency of risk factors between the VOD/SOS and non-VOD/SOS groups; the VOD/SOS group is younger, and as expected most of them are affected with advanced-stage disease and received myeloablative conditioning. Conjugated antibody therapy with inotuzumab or gemtuzumab had previously been administered in two patients. Compared to the non-VOD/SOS groups, none had had previous liver impairment or a second allogeneic haematopoietic stem cell transplant.

**Table 2:** Differences between the VOD and non-VOD patients.

**Risk factors present**	**VOD/SOS cases (*N* = 12) *N* (%)**	**NON-VOD/SOS patients (*N* = 98) *N* (%)**
Previous gemtuzumab or inotuzumab	2 (16)	7 (7)
Iron overload (ferritin > 500 ug/L)	5 (42)	57 (58)
Age > 65	0 (0)	25 (25)
Performance status (Karnofsky score) <90%	2 (16)	19 (19)
Metabolic syndrome	2 (16)	13 (13)
Norethisterone	3 (25)	7 (7)
Advanced disease^+^	6 (50)	36 (37)
Unrelated donor	11 (92)	73 (74)
HLA-mismatched*	7 (59)	36 (37)
MAC	10 (83)	31 (32)
High dose Bu	9 (75)	29 (30)
High dose TBI	1 (8)	2 (2)
Transaminasis > 2.5 ULN	0 (0)	6 (6)
Bilirubin > 1.5 ULN	0 (0)	2 (2)
Second allograft	0 (0)	11 (11)

^*^it counts mismatched unrelated donors and haploidentical sibling donors; + beyond second CR or active disease; patients with molecular marker positive before transplant are not considered except BCR-ABL > 1% IS. The table shows frequencies of risk factors in the two groups of patients.

### VOD/SOS characteristics and treatment

Twelve patients were diagnosed with severe VOD/SOS at a median of 22 days (range: 8–44) following HSCT; the median age of this group of patients was 51 (range: 30–65). Late-onset VOD/SOS was diagnosed in 45% of our patients in a median time of 38 days (range 29–44). No cases of anicteric VOD/SOS were diagnosed.

The treatment with defibrotide started when the patient met the diagnostic criteria for severe VOD at a median of 14 days after HSCT (range: 8–44); three patients were diagnosed at the VOD/SOS ward round and started treatment within the same day. The remaining patients were highlighted to the ward attending team by the VOD/SOS ward round team due to the presence of multiple risk factors and progressive mild increase in body weight or bilirubin level; this information triggered increased monitoring by ward nurses if not already taking place and allowed a daily evaluation for VOD/SOS from the ward attending team. Nine patients were diagnosed with severe VOD/SOS in a median time of 3 days after the weekly evaluation (range: one to six) and started treatment on the same day.

Before the implementation of this protocol, the review of cases from 2018 to 2020 demonstrated that the median time to commence defibrotide treatment for VOD/SOS was 2 days (range 0–81) following initial VOD/SOS symptoms. The rigorous evaluation of diagnostic criteria and awareness of the risk factors present has subsequently led to a 48-h reduction in starting the treatment compared to those diagnosed during the 2 years prior.

All diagnosed patients had an ultrasound of the abdomen that documented hepatomegaly and ascites. Two patients also underwent a CT scan of the abdomen that confirmed the findings and ruled out other conditions with similar symptoms, such as obstructive jaundice or typhlitis.

Only one patient, despite the treatment and high-intensity supportive care, was escalated to the intensive care unit and subsequently died due to multiorgan failure. The remaining patients achieved complete resolution of the complication with 21 days of defibrotide treatment and enhanced care as described above.

### Practical impacts of the VOD/SOS ward round

The dedicated VOD/SOS ward round was able to highlight that most of our transplant recipients had multiple risk factors for this complication.

This strategy offered regular evaluation and provided documentation (at least once a week) relating to the complication in the patient's electronic notes. Since September 2020, the median number of VOD/SOS ward round entries in the clinical notes are three (range: 1–25); these multiple entries are a consequence of long admission or re-admissions. Because of the written documentation, we can evidence our systematic and consistent approach to the monitoring and diagnosis of VOD/SOS which contrasts with the ad hoc approach used previously.

Furthermore, the weekly evaluation and documentation promoted discussion of the findings at our multidisciplinary meetings and at the handover meeting before weekends and bank holidays, and has been incorporated into future planning of patient care. It is clear that the impact of the ward round extends beyond the weekly event, with quicker diagnosis of this complication due to increased awareness and confidence within the wider team.

## Discussion

VOD/SOS can be a devastating complication of HSCT if not diagnosed and treated promptly. A recent retrospective EBMT study showed that VOD/SOS was misdiagnosed or not recognized at all in nearly 69% of transplant patients who died with a diagnosis of multi-organ failure syndrome (7).

The insidious onset of VOD/SOS and the presence of several confounding issues in the early stage of HSCT (such as sepsis, drug induced liver injury, graft vs. host disease) can lead to a delay in diagnosis and treatment, hence prompt identification of patients at risk can be lifesaving (8, 9). Overall, the majority of transplant patients assessed in the ward rounds were noted to be at risk of VOD/SOS, with 97% of patients having undergone HSCT with at least one risk factor present.

Our transplant program is myeloid focused with a large referral practice for high-risk myeloid neoplasms and bone marrow failure syndromes. This likely contributes to the high numbers of patients with advanced-stage disease, iron overload, and reduced organ performance status, which may explain the relatively higher incidence of severe VOD/SOS in our study.

Another possible explanation for the higher incidence is a consequence of our ability to diagnose late-onset VOD/SOS.

Additionally, the systematic evaluation and documentation of risk factors allowed us to identify patients that developed severe VOD/SOS because these patients had at least three risk factors present. Compared to our previous experience (10), the reduction in time to treatment of 48 h most likely avoided the onset of irreversible critical damage to the endothelium (11), and this may explain the near absence of ICU admission and death in the reported cohort.

Even though VOD/SOS is commonly recognized as a busulfan- or radiotherapy-related toxicity (12, 13), alloreactivity within an unhealthy Disse space remains an important factor, and for this reason the extra level of attention to a recipient of mismatched allograft or MAC is warranted (14, 15).

It is important to highlight that the focused ward round acts as a safety net, by raising the awareness of hospital staff to patients at higher risk of the onset of this complication, helping trigger early investigations for prompt diagnosis, and raising the profile of this rare complication within our clinical setting, as well as offering educational value for both our nursing and junior medical teams.

The focused evaluation from a doctor and nurse allows careful review of vital signs, assessment of physical examination findings, and drug chart and laboratory evaluations. This enabled propagation of the findings to the ward-attending medical and nursing teams, allowing more intense monitoring and appropriate investigations in patients suspected to be developing VOD/SOS.

Indeed, awareness of this complication along with bedside identification of patients at risk of VOD/SOD allowed the daily ward team to evaluate carefully and monitor the patients daily, which avoided delays in diagnosis.

Our study showed that focused monitoring of risk factors and regular evaluations led to generally different risk factors for VOD/SOS being disclosed for the transplant population. Due to the rarity of this complication and the difficulty of reproducing it in experimental models, the hierarchy of risk factors for VOD/SOS is still poorly understood. It is still too early to confirm whether the dedicated ward round is a sufficiently informative instrument to clarify which risk factors are most important within an *in-vivo*, T-cell-depleted transplant program like ours. In contrast to the EASIX-score (16), this specialized evaluation is a regular bedside assessment of the patient, combined with consideration of risk factors present and physical examination findings; however, it is important to highlight that VOD/SOS as a transplant complication is a dynamic entity, and the current VOD/SOS ward round still lacks either a blood parameter value or a serological biomarker to further classify the patient after stem cell infusion or to diagnose this complication before the onset of raised bilirubin.

In conclusion, the focused VOD/SOS evaluation demonstrates that our transplant population is generally at risk of VOD/SOS because of different risk factors. The early identification of patients with multiple risk factors for VOD/SOS allowed diagnosis of the complication before the onset of multiorgan failure, and we were able to decrease the median time to treatment by 48 h. A formal VOD/SOS ward round was fundamental to document all the risk factors and is likely to have educational value for the health care professionals within the BMT/HSCT service.

## Data availability statement

The original contributions presented in the study are included in the article/supplementary material, further inquiries can be directed to the corresponding author.

## Ethics statement

The studies involving human participants were reviewed and approved by King's College Hospital. The patients/participants provided their written informed consent to participate in this study.

## Author contributions

DA and MK designed the ward round, collected the data, and analysed and wrote the manuscript. AP and VP reviewed and supervised the data analysis and the manuscript. VM, PK, AK, SG, FD, MN, HW, YL, AE, SA, MCu, MCo, CB, DD, PH, and AP contributed in writing the manuscript and in patients care. All authors contributed to the article and approved the submitted version.

## Conflict of interest

The authors declare that the research was conducted in the absence of any commercial or financial relationships that could be construed as a potential conflict of interest.

## Publisher's note

All claims expressed in this article are solely those of the authors and do not necessarily represent those of their affiliated organizations, or those of the publisher, the editors and the reviewers. Any product that may be evaluated in this article, or claim that may be made by its manufacturer, is not guaranteed or endorsed by the publisher.
